# Relative Influence of Plastic Debris Size and Shape, Chemical Composition and Phytoplankton-Bacteria Interactions in Driving Seawater Plastisphere Abundance, Diversity and Activity

**DOI:** 10.3389/fmicb.2020.610231

**Published:** 2021-01-13

**Authors:** Jingguang Cheng, Justine Jacquin, Pascal Conan, Mireille Pujo-Pay, Valérie Barbe, Matthieu George, Pascale Fabre, Stéphane Bruzaud, Alexandra Ter Halle, Anne-Leila Meistertzheim, Jean-François Ghiglione

**Affiliations:** ^1^UMR 7621, CNRS, Laboratoire d’Océanographie Microbienne, Observatoire Océanologique de Banyuls-sur-Mer, Sorbonne Université, Banyuls-sur-Mer, France; ^2^Génomique Métabolique, Genoscope, Institut François Jacob, CEA, CNRS, Univ Evry, Université Paris-Saclay, Evry, France; ^3^Laboratoire Charles Coulomb (L2C), UMR 5221 CNRS-UM, Place Eugène Bataillon, Montpellier, France; ^4^Institut de Recherche Dupuy de Lôme (IRDL), Université Bretagne Sud, UMR CNRS 6027, Lorient, France; ^5^IMRCP, Université de Toulouse, CNRS, Toulouse, France; ^6^SAS Plastic@Sea, Observatoire Océanologique de Banyuls-sur-Mer, Banyuls-sur-Mer, France

**Keywords:** plastic litter, plastisphere, biofilm, biofouling, colonization, microbial ecotoxicology

## Abstract

The thin film of life that inhabits all plastics in the oceans, so-called “plastisphere,” has multiple effects on the fate and impacts of plastic in the marine environment. Here, we aimed to evaluate the relative influence of the plastic size, shape, chemical composition, and environmental changes such as a phytoplankton bloom in shaping the plastisphere abundance, diversity and activity. Polyethylene (PE) and polylactide acid (PLA) together with glass controls in the forms of meso-debris (18 mm diameter) and large-microplastics (LMP; 3 mm diameter), as well as small-microplastics (SMP) of 100 μm diameter with spherical or irregular shapes were immerged in seawater during 2 months. Results of bacterial abundance (confocal microscopy) and diversity (16S rRNA Illumina sequencing) indicated that the three classical biofilm colonization phases (primo-colonization after 3 days; growing phase after 10 days; maturation phase after 30 days) were not influenced by the size and the shape of the materials, even when a diatom bloom (*Pseudo-nitzschia* sp.) occurred after the first month of incubation. However, plastic size and shape had an effect on bacterial activity (^3^H leucine incorporation). Bacterial communities associated with the material of 100 μm size fraction showed the highest activity compared to all other material sizes. A mature biofilm developed within 30 days on all material types, with higher bacterial abundance on the plastics compared to glass, and distinct bacterial assemblages were detected on each material type. The diatom bloom event had a great impact on the plastisphere of all materials, resulting in a drastic change in diversity and activity. Our results showed that the plastic size and shape had relatively low influence on the plastisphere abundance, diversity, and activity, as compared to the plastic composition or the presence of a phytoplankton bloom.

## Introduction

Plastic pollution has become a global environmental problem affecting all parts of oceans worldwide, including the most remote areas such as deep seafloor or polar regions. In this area, the longevity of the plastics is estimated to be hundreds to thousands of years (Barnes et al., 2009; Lusher et al., 2015; Kane et al., 2020). Vast accumulation zones have been identified in the five subtropical oceanic gyres (Van Sebille et al., 2015), but also in the Mediterranean Sea that has been proposed as the sixth great accumulation zone for marine litter (Cózar et al., 2015). Variation in quantity and composition was observed throughout the different environmental compartments: polyethylene (PE) and polypropylene (PP) were mostly observed in epipelagic waters, whereas polyamide and polyester dominated in sediments. These variations have been explained through the differences in density, surface area, and the size of plastic litter (Chubarenko et al., 2016; Kowalski et al., 2016; Schwarz et al., 2019).

Once entering the environment, plastic litter is subjected to degradation caused by a combination of mechanical abrasion, photo- or thermal-oxidation, hydrolysis and biodegradation (Andrady, 2003). Plastic degradation results in the formation of tiny plastic fragments of <5 mm size, so-called “secondary microplastics” that differ from the “primary microplastics” that are designed and produced as purpose, for example in industrial cleaners and personal care products. Larger plastics are classically categorized into meso-debris (5 mm–2 cm) and macro-debris (>2 cm) for large-scale and long-term monitoring of plastic litter across countries and environments (Thompson et al., 2009). Over the estimated 5.25 trillion particles are afloat in the global ocean, 34.8% of which are small microplastics (SMP; 330 μm–1 mm), 57.5% of large microplastics (LMP; 1–5 mm), 7% of meso-plastics, and 0.2% of macro-plastics (Eriksen et al., 2014). Large debris have been shown to have adverse effects on fish, seabirds, and other top consumers, whereas microplastics are suitable for ingestion by smaller organisms at lower trophic levels (Wang et al., 2019).

When directly released at sea, plastics are primarily colonized by microorganisms that form dense biofilms on their surfaces, the so-called “plastisphere” (Zettler et al., 2013). The plastisphere has multiple effects on the fate and impacts of plastic in the marine environment. First, the biofilm growing on the surface and inside plastic cracks can contribute to a loss of physical integrity, a phenomenon called “biodeterioration” that play a significant role on the breakdown of large plastic debris into microplastics when coupled with abiotic degradation (Sabev et al., 2006; Dussud and Ghiglione, 2014). Second, the biofouling may increase or decrease the buoyancy of the plastic particles, rendering them susceptible to vertical transport (Kooi et al., 2017; Kane et al., 2020). Third, extracellular polymeric substances produced by the biofilm contribute to co-aggregation of microorganisms and detritus together with microplastics, thus resulting in an increase or decrease of sedimentation rates of algal bloom (such as diatoms or cryptophytes) with important impact on ecosystem functioning (Long et al., 2015; Severin et al., 2017). Fourth, biofilms alter the physico-chemical properties of plastics and increase further colonization by metazoan larvae (Hadfield, 2011; Ghiglione and Laudet, 2020). Fifth, biofilms can host pathogens species that can be transported across the marine environment by plastic dispersion and thus participate to the diffusion of infectious diseases (Zettler et al., 2013; Kirstein et al., 2016). And finally, plastic biodegradation is promoted by the biofilm by secreting extracellular enzymes able to transform polymers into oligomers and monomers (“biofragmentation”), which can serve as carbon source for microbial growth (“bio-assimilation”) that may result in the complete mineralization of polymers into CO_2_ and H_2_O (“biomineralization”) (Jacquin et al., 2019).

A growing literature is reporting the large diversity of microorganisms that composed the plastisphere, which differed from the surrounding communities living in a free-living state (Zettler et al., 2013; Bryant et al., 2016; Pinto et al., 2019), or attached to organic particles (Dussud et al., 2018b), sediment particles (Basili et al., 2020) or other substrates such as wood, cellulose or glass (Kirstein et al., 2018; Oberbeckmann et al., 2018; Ogonowski et al., 2018). The reasons for the preferential attachment of specific communities to plastic particles is still enigmatic. Within the plastisphere communities directly sampled at sea, several factors such as plastic type (polyethylene, polypropylene, polystyrene), geographical location or seasons appeared to differentiate the biofilm communities (Amaral-Zettler et al., 2015; Oberbeckmann et al., 2018). Other factors such as hydrophobicity, topography, roughness, crystallinity, and surface charge may play a role in the selection of bacterial community in the early stages of colonization, which is a crucial step for the following colonizing communities by modifying the material-specific surface properties (Rummel et al., 2017). Most of the above studies focused on microbial diversity and abundance, but only one evaluated the corresponding activity of the microorganisms that form the biofilm (Dussud et al., 2018a). Moreover, only one observation based on field study tested the influence of plastic size and shape (Frère et al., 2018) that is often mentioned as having a crucial role in shaping the biofilm (Oberbeckmann et al., 2015; Harrison et al., 2018), but no specifically designed experiment was dedicated to this question so far.

The aim of this study was to test how much plastisphere was influenced by different polymer composition (PE and PLA), sizes (SMP, LMP, and meso-plastics of 100 μm, 3 mm, and 1.8 cm in diameter, respectively) and shape (spherical vs. irregular in the case of SMP). During the 2-months incubation in natural seawater from the NW Mediterranean Sea, we also evaluated the impact of environmental change such as a phytoplankton bloom. We hypothesized that the plastisphere may be more influenced by phytoplankton-bacteria interactions rather than the plastic composition, size or shape. Temporal variations of bacterial abundance (confocal microscopy), diversity (16S rRNA sequencing) and heterotrophic activity (radiolabeled leucine incorporation) were measured on all plastic types, but also compared to glass of similar size and shape, as well as to the surrounding seawater.

## Materials and Methods

### Preparation of Polymers of Different Composition, Size and Shape

High density-polyethylene (HDPE) and Poly L lactic acid (PLA) were supplied by the Good Fellow company (Avilés, Spain) in a form of film of 10 and 50 μm thickness (± 20%), respectively. Circular pieces of respectively 3 mm and 1.8 cm in diameter were cut using a hole puncher. Glass coverslip (soda lime composition) were supplied by the Verres Vagner company (Toulouse, France) in circular form with 1.8 cm and 3 mm diameter of 170 μm thickness.

Irregular PLA and glass microbeads were obtained by cryo-grinding the polymer and glass films described above (SPEX sample Prep), which were further sieved with ethanol in order to recover the microparticles for which the size was ranging from 90 to 125 μm. Material for HDPE irregular microbeads were obtained from Good Fellow films with 1 mm thickness to ensure the 3-dimensional structure, and then reduced in size by cryo-grinding as described above.

Spherical HDPE microbeads of size distribution between 96–125 μm were commercially available (CPMS-0.96, Cospheric^TM^). Spherical PLA microbeads were obtained as pellets and transformed in spherical microbeads by solvent emulsion-evaporation technique (O’Donnell and McGinity, 1997). It consisted in dissolving the polymer in a volatile organic solvent immiscible with water (dichloromethane), then introducing this solution into an aqueous solution containing an emulsifier as poly(vinyl alcohol) (PVA, 2%). The emulsion was finally placed under moderate magnetic stirring for 24 h at atmospheric pressure and ambient temperature, in order to allow the microbeads to harden, until complete evaporation of the organic solvent. The spherical PLA microbeads were collected by wet sieving between 90 and 125 μm, rinsed with permuted water and lyophilized until further use. Spherical glass microbeads were mainly made up with soda lime and commercially available (Good Fellow company, Avilés, Spain).

Granulometry analysis using a laser diffraction particle size analyzer (Mastersizer 2000 model with a Scirocco 2000 module, Malvern, United Kingdom) showed a gaussian distribution of the microbeads that always peaked at 100 μm for all polymers for spherical or non-spherical beads. Before the experiment, all the materials (including irregular microbeads (IR), spherical regular microbeads (RE) of average 100 μm diameter as well as films of 3 mm and 1.8 cm in diameter) were washed for 1 h with ethanol followed by 3 rounds of vortex (1 min) and sonic bath (3 min) and then dried under the sterile hood.

### Experiment Setup

Each material type (spherical or irregular 100 μm microbeads, 3 mm and 1.8 cm films of PE, PLA and glass, respectively) was placed separately in 12 identical glass tanks of 2 L capacity (Plastic@Sea, Banyuls-sur-mer, France), in which seawater was continually renewed (flow rate was set on 20 mL min^–1^) by direct pumping at 14 m depth in Banyuls bay closed to the SOLA observatory station (NW Mediterranean Sea, France). Three additional tanks containing circulating seawater served as controls. Seawater was pre-filtered with 20 μm porosity filters (Dutscher^TM^, France) to remove inorganic matter and potential predator in front of each tank. The tanks were placed in a dark room and illuminated from above in a 12/12 h light/dark rhythm by Lumivie LED RAL G2-SBM lamps (Zoomalia, France) with a nominal luminous flux of 1860 lm each. The experiment started from 13 August 2019, and samples were taken after 3, 10, 30, and 66 days (designated as D3, D10, D30, and D66, hereafter).

### Seawater Environmental Variables

Temperature, salinity, inorganic nutrients, chlorophyll *a*, and particulate organic carbon were weekly recorded *in situ* at the SOLA station (0.5 miles off the coast) in the framework of the French national coastal monitoring program “Service d’Observation en Milieu Littoral” (SOMLIT) according to protocols previously described (Ghiglione et al., 2005; Blanchet et al., 2017) and available on the SOMLIT website^1^. All samples were processed after sampling within 30 min.

### Confocal Microscopy and Flow Cytometry

For each sampling date, triplicate samples were fixed with 1% (v/v) glutaraldehyde for 30 min before freezing until analysis. Samples were stained with a 4,6-diamidino-2-phenylindole (DAPI) solution (final concentration 10% [v/v], Sigma Aldrich, France) for at least 5 min in the dark at ambient temperature before confocal microscopy observations (TCS SP8 confocal laser scanning microscope, Leica, Germany). Photomultiplier tubes (PMT3) detector was used for detecting the fluorescence signal and transmitted light detector (TLD) was used to capture the white light signal. The light intensity was compensated for the microbeads, and the Z-Step size were set on 1 μm to get regularly spaced cross sections. For each sample, 3 beads or 3 pieces of films were used for counting the bacterial abundance using the image J software (Abràmoff et al., 2004). For the regular spherical microbeads, the surface area was calculated using a simple geometrical formula. For irregular microbeads, the surface area was estimated using two different methods. First, a simple geometrical calculation based on the overall shape of the particle, i.e., ellipsoidal, cylindrical or conic. The main dimensions used for the surface area calculation were measured from optical images. In addition, for a series of samples, the surface was calculated via a full reconstruction of the particle surface using 1 μm separated confocal microscopy cross-sections, using the image-J software. A 10% agreement was found between the two methods (geometrical estimation and 3D volume reconstruction) for the samples studied, thus validating the use of a simple geometrical method for irregular beads and giving us the surface measurement uncertainty. Cell counts were verified using the Gwyddion software (Neèas and Klapetek, 2012) threshold filter and grains numbering. Manual counting was performed on a series of samples to double check the cell counts accuracy, which was found to be of 10%. Cell counts were then expressed as the number of cells over surface area (in cells mm^–2^) with an accuracy of 20%.

In parallel, 1 mL of seawater from the control tank was also fixed using the same procedure. A volume of 500 μL control seawater was mixed with the nucleic acid dye SYBR Green I (final concentration 0.05% [v/v], Sigma Aldrich) for 15 min at room temperature in the dark. Cell counts were performed with a FACSCanto II flow cytometer (BD Bioscience, San Jose, CA, United States) equipped with a blue laser (488-nm, air-cooled, 20-mW solid state), as previously described (Mével et al., 2008).

### Heterotrophic Bacterial Production

Bacterial production was measured in triplicate for each material at each sampling time by ^3^H-leucine incorporation (Dussud et al., 2018a). In brief, the films with the size of 3 and 18 mm were rinsed with sterile filtered seawater using a wash bottle before transferring to the microtubes containing 1.5 mL sterile filtered seawater (0.2 μm pore size, polycarbonate filter, Nucleopore). Microbeads were collected on a membrane filter with 10 μm pore size membrane filter (LCWG02500, Mitex^TM^) and then rinsed with sterile filtered seawater, and the seawater was removed by air pumping for 30 s to get accurate sample weight. Different mass of materials (15 mg for PE, 18 mg for PLA, and 33 mg for glass microbeads) were collected in order to homogenize the total surface areas, and then transferred into microtubes before adding 1.5 mL of sterile filtered seawater. A cell detachment pre-treatment was applied for all the materials using 3 cycles of 3 min sonication bath (Delta Sonic, France) followed by 3 min vortex at maximum speed (Skyline, Elmi Ltd., Russia). Immediately after cell-detachment, ^3^H-leucine (125.6 Ci mmol^–1^, Perkin Elmer^TM^) were added at 1 nmol L^–1^ final concentration (completed with cold leucine to 150 nmol L^–1^), which consisted of 1.5 ml sterile seawater containing the film or microbeads and detached bacteria. For seawater samples from the control tanks, ^3^H-leucine was added at a final concentration of 4.3 nmol L^–1^ to 1.5 mL of control seawater. All the samples were incubated in the dark at 18°C for 3 h. The empirical conversion factor of 1.55 ngC pmol^–1^ of incorporated leucine was used to calculate the bacterial heterotrophic production (Kirchman, 2001; Céa et al., 2015). For each microbeads on each sampling date, the percentage of hydration of the beads were assessed by weighting 3 additional aliquots (wet weight), that were frozen, lyophilized and weighted a second time to measure dry weight. Knowing the wet weight and the percentage of hydration of each material during the kinetic, the bacterial activity was expressed per dry weight.

Knowing the number of cells (N) per unit area for each sample and the average specific surface R_SV_ (i.e., surface to volume ratio) for each type of microbeads, we expressed the heterotrophic bacterial production as the carbon produced per unit area per unit time (ngC mm^–2^ h^–1^) or the specific bacterial activity as the carbon produced per cell per unit time (fgC cell^–1^ h^–1^). This data representation allows to meaningfully compare materials having different shapes, i.e., different surface to volume ratios.

Additionally, we used bacterial production data derived from leucine incorporation to estimate the bacterial growth rate on plastisphere, using a conversion factor of 12 fgC per cell as previously described (Fukuda et al., 1998).

### DNA Extraction, PCR, Sequencing and Data Analysis

On each sampling date, triplicates of plastic or glass were harvested from each sample by using the same method described above for the bacterial production and immediately stored at −80°C. Triplicates of 2 L seawater from control tanks were also obtained using 0.2 μm pore size polycarbonate filters (47 mm diameter, Nucleopore). The microbial genomic DNA extraction were followed with classic phenol-chloroform protocol (Ghiglione et al., 1999; Marty et al., 2012) and DNA was quantified by DeNovix (DS-11 series, United States). Polymerase chain reaction (PCR) amplification was done using universal small subunit ribosomal RNA (SSU rRNA) primers (515Y, ^5^Parada et al., 2016; Yeh et al., 2019). Illumina MiSeq sequencing were performed at Genoscope (Evry, France) for the 156 samples, corresponding to the 144 samples of PE, PLA and glass (3 substrate ^∗^ 4 sampling date ^∗^ 4 size fraction ^∗^ 3 replicates) and the 12 seawater samples (4 sampling date ^∗^ 3 replicates). All the SSU rRNA data are available in the NCBI SRA repository (accession number PRJNA663787).

Processing of SSU rRNA sequences was performed using the DADA2 R package (Callahan et al., 2016). Forward and reverse 16S reads were trimmed off before error correction and denoising step. Paired reads were merged (average length from 367 to 377 bp) and all the singletons and chimeras were removed. 18S reads are 575–595 bp, which is too long for forward and reverse reads to overlap. As recommended by Parada et al. (2016) and Yeh et al. (2019), we trimmed the 18S forward reads to fixed length (220 bp) and the reverse reads was discarded before error correction and denoising step. Denoised reads were concatenated and chimeric reads were discarded. The 16S and 18S amplicon sequence variants (ASVs) were assigned with SILVA 128 database (Quast et al., 2013). The taxonomic affiliation of ASVs of interest were further verified against sequences from the NCBI database using the BLASTnt. The ASVs corresponding to eukaryotes, archaea, chloroplast and mitochondria were removed and all the samples were rarified to the same number (rngseed = 1) before the analyses on bacteria using the phyloseq R package (McMurdie and Holmes, 2013). The α-diversity was performed using the *alpha()* function from the microbiome R package. Square root transformation was performed for the β-diversity analyses and hypotheses test. The taxonomy compositions were visualized using the histogram and bubble plot with the ggplot2 R package (Wickham, 2016).

To investigate the unique and shared bacterial community between biofilm and seawater, the reads from plastic or glass with different size fractions were pooled and rarefied into the same number to that of seawater. Two levels of comparison were conducted: in ASV level (presence or absence) and in tags levels.

### Statistical Analyses

An unweighted-pair group method with arithmetic (UPGMA) dendrogram based on Bray-Curtis similarities was used for visualization of beta-diversity. A similarity profile test (SIMPROF, PRIMER 6) was performed on the null hypothesis that a specific sub-cluster can be recreated by permuting the entry species and samples. The significant branch was used as a prerequisite for defining bacterial cluster.

The significance of the factor size (including irregular and regular microbeads), the substrate and the date were analyzed using the global or pairwise permutational multivariate analysis of variance (PERMANOVA) (Anderson and Walsh, 2013) using the *adonis()* function with the vegan R package (Oksanen et al., 2007), the homogeneity of variances was tested using the *betadisper()* function. The *p* value was adjusted with the Benjamin-Hochberg method. To test the bacterial community relationship between seawater and plastic, a Mantel test was performed in Vegan using the *mantel()* based on Pearson correlation method.

Three-way analyses of variance (ANOVA) were performed for statistical analyses with the type I sum of square applied for the results on bacterial abundance, bacterial production, bacterial activity and bacterial growth rate. Tukey’s *post hoc* test was used when necessary. Relative importance of each predictor (in R square) for ANOVA results was further determined with function of *calc.relimp()* from relaimpo R package (Grömping, 2006). When the data did not meet homogeneity, the Welch’s ANOVA was chosen for the analyses of α-diversity and the ASVs comparison on results of the bubble plot. Games-Howell test was used as *post hoc* test.

## Results

### Environmental Conditions

During the studied period (13th August – 20th October), the environmental conditions in the Bay of Banyuls were characteristic of an autumn situation in a temperate Mediterranean area. The surface water temperature remained stable (from 20 to 23°C) as well as the salinity that stayed around 38 (Supplementary Figure 1A).

As expected in late summer, concentrations of silicate were low (∼0.5 μM) or often close to the detection limit for nitrate and phosphate (<0.05 μM and <0.02, respectively) (Supplementary Figure 1B). Two events were clearly identified during the period and marked by a nutrient enrichment in the water column. The first had a limited magnitude on 24th September and the second was more important at the very end of the experiment on 25th October with concentrations of 2.4, 0.93, and 0.18 μM in Si(OH)_4_, NO_3_ and PO_4_, respectively.

These nutrient inputs due to the first mixing inducing the disruption of the water column stratification were responsible for an increase in particulate matter in the water column as shown in Supplementary Figure 1C in terms of Total Suspended Matter (TSM), and especially in terms of chlorophyll. After a low and homogeneous concentration during August and the beginning of September (range between 0.1 and 0.2 mg m^–3^ of chlorophyll a), we observed 2 peaks (0.5 and 0.6 mg m^–3^ of chlorophyll a) characteristic of a coastal autumn bloom situation.

### Microbial Cell Counts and Shape

**FIGURE 1:**
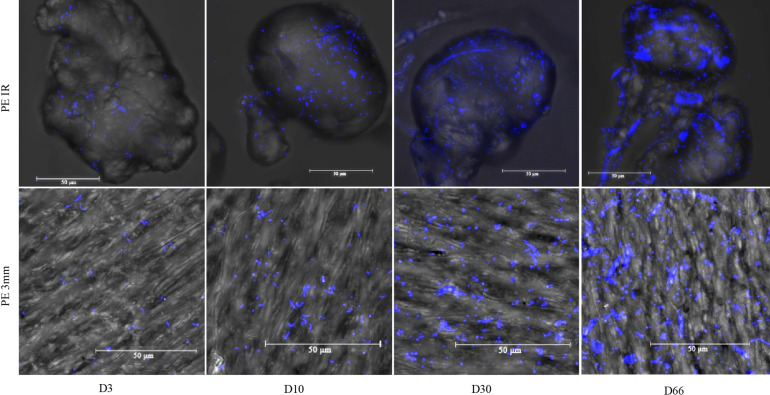
Confocal microscopy for polyethylene irregular microbeads of around 100 μm diameter (PE IR) and 3 mm film (PE 3 mm) at days 3 (D3), 10 (D10), 30 (D30), and 66 (D66). Scale bar: 50 μm. Arrows are pointing typical shape of diatoms that appeared at D66 only.

Confocal microscopy revealed a large diversity of morphological forms including spherical, rod-shaped or spiral-shaped bacterial like structure at the surface of PE, PLA and glass, for which rod-shaped and spiral-shaped structure was more observed on D3 and D10 compared to D30 and D66 (Figure 1). Typical morphotypes of diatoms appeared at D66 and were not visible before. Confocal microscopy was also useful to confirm the size distribution of microbeads between 90 and 125 μm, as well as their shape (regular versus irregular).

**FIGURE 2:**
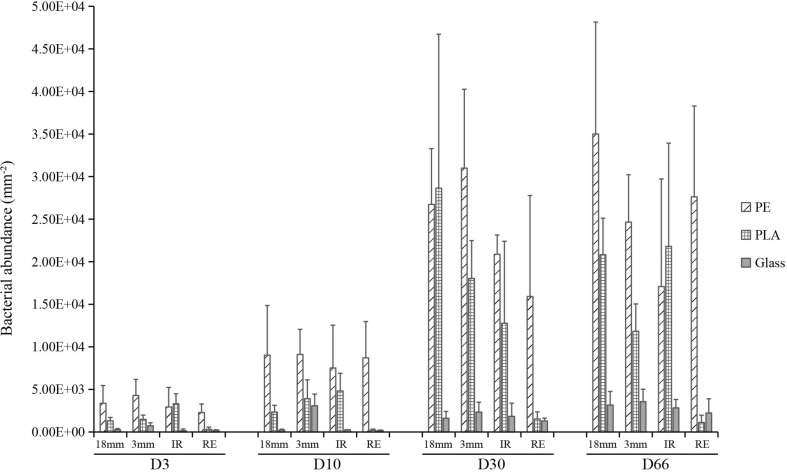
Bacterial cell counts per surface area (mm^– 2^) for PE, PLA and glass on mesoplastics (18 mm diameter), large microplastics (3 mm diameter) and small microplastics microbeads (100 μm diameter) with irregular (IR) or regular spherical shapes (RE) during the course of the experiment at days 3 (D3), 10 (D10), 30 (D30), and 66 (D66).

Triplicate samples analyzed by confocal microscopy allowed us to follow the changes in bacterial counts for each material type and size. The data highlighted three distinct phases of biofilm formation: primo-colonization, growth, and maturation (Figure 2). Interestingly, the three distinct phases were found whatever the material type or size. Three-way ANOVA revealed significant difference in bacterial counts according to the sampling date and material type, but not within material sizes (material sizes containing the two shapes of IR and RE) (*R*^2^ = 0.29, 0.29, and 0.01, respectively). PE presented the largest bacterial abundance on average together with PLA, whereas it appeared to be ten-fold smaller on glass. A rapid primo-colonization was observed after D3, with average abundance of 3.0 × 10^3^, 1.6 × 10^3^, and 0.3 × 10^3^ cells mm^–2^ for PE, PLA and glass, respectively. Slight growth was observed between D3 and D10 for PE, PLA and glass, where the bacterial abundance for glass samples remained relatively low (8.6 × 10^3^, 2.8 × 10^3^, and 0.9 × 10^3^ cells mm^–2^, respectively). We observed a significant increase in bacterial abundance between D10 and D30. Since we did not find significant increase in bacterial counts at a later time point (between D30 and D66, *p* > 0.05), it indicates that the maturation phase corresponding to the stabilization of bacterial abundance was reached for all material types from D30 and until the end of the experiment (D66). On average the mature biofilm was of 2.5 × 10^4^, 1.5 × 10^4^, and 0.2 × 10^4^ cells mm^–2^ for PE, PLA and glass, respectively, with non-significant differences between size and shapes within each material type. It should be noted that PLA regular microbeads was excluded from the statistical analysis for bacterial abundance and activity, because we noted a significantly lower abundance compared to other PLA samples, which was much closer to those of the glass samples. Indeed, we realized that the use of surfactants in the home-made synthesis of PLA regular beads could be responsible for this behavior, as surfactant could be able to remain at the beads surfaces and considerably modify their hydrophobicity.

Diatoms with the average length around 11 μm were observed only on D66 on all materials, with higher abundance for 3 mm and 1.8 cm sizes on PE (3.5 × 10^3^ cells mm^–2^) and PLA (2.4 × 10^3^ cells mm^–2^) compared to glass (0.8 × 10^3^ cells mm^–2^). They were rarely visible on spherical or regular microplastics.

The bacterial counts from seawater controls remained stable from 4.5 × 10^4^ to 1.0 × 10^5^ cells per mL all along the experiment.

### Heterotrophic Bacterial Production

**FIGURE 3:**
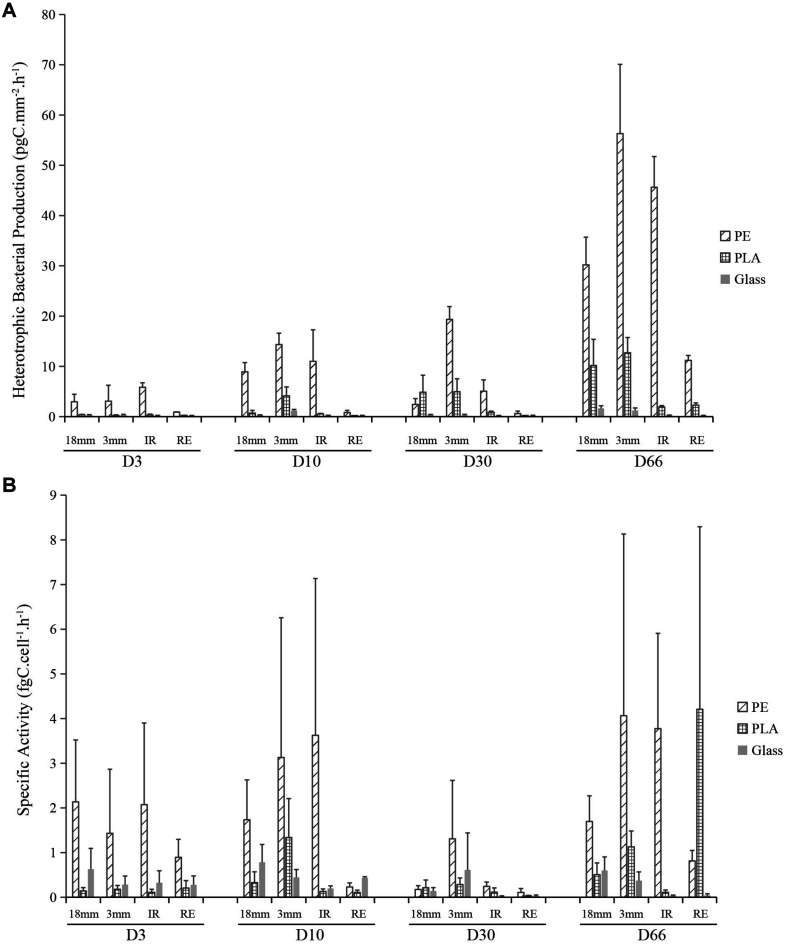
Bacterial heterotrophic production (BP, pgC mm^– 2^ h^– 1^) **(A)** and bacterial activity (fg C cell^– 1^ h^– 1^) **(B)** for PE, PLA and glass on larger pieces (18 mm diameter), microplastics (3 mm diameter) and irregular (IR) or regular spherical (RE) microbeads (100 μm diameter) during the course of the experiment at days 3 (D3), 10 (D10), 30 (D30), and 66 (D66).

Heterotrophic bacterial production (BP) was measured alongside the time course of the experiment and it was expressed in units of incorporated carbon per square millimeter per hour (pgC mm^–2^ h^–1^) (Figure 3A). Three-way ANOVA showed that all the factors (sampling date, material type, and size) could significantly impact the BP, being the sampling date and material type factors explaining more variation than the material size (material sizes containing the two shapes of IR and RE) (*R*^2^ = 0.20, 0.25 and 0.07, respectively). Significant increase has been observed from the primo-colonization phase (on D3) to the growing phase (on D10). Interestingly, the BP did not show significant differences between D10 and D30, even though there was a significant increase in bacterial abundance (Figure 2). The maturation phase showed a significant increase of BP from D30 to D66. Differences in BP were also observed on the three sample types, with PE being significantly higher than PLA and glass. In average, BP of PE is 16 and 110 times higher than that of PLA and glass by comparing within each material size. The BP of PE, PLA and glass have increased from 3.2, 0.2, and 0.1 pgC mm^–2^ h^–1^ at the primo-colonization phase (D3) to 35.8, 6.8, and 0.7 pgC mm^–2^ h^–1^ at the maturation phase (D66), with intermediate maturation phase showing 6.8, 2.7, and 0.1 pgC mm^–2^ h^–1^, respectively (D30). Besides, BP difference from material type was also observed, with LMP of 3 mm size being higher than the rest of the samples, and the regular SMP microplastics of 100 μm size presenting the lowest values. Generally, PE and glass in regular SMP were 11 and 6 times lower than other material sizes (also containing form). PLA regular SMP were 150 times lower than other sizes or shape. The BP for seawater ranged from 88 to 150 pgC mL h^–1^ without significant difference between sampling dates.

Bacterial specific activity was further calculated by dividing the BP by the bacterial abundance in the unit of incorporated carbon per cell per hour (fgC cell^–1^ h^–1^). Three-way ANOVA also showed that all the factors (sampling date, material type, and size) could significantly impact the specific activity, the material type explaining more variation than the sampling date and material size (material sizes containing the two shapes of IR and RE) (*R*^2^ = 0.08, 0.27, and 0.07, respectively). Interestingly, BP on the primo-colonization phase of D3 and growing phase of D10 and maturation phase of D66 were significantly higher than during the maturation phase at D30 (*p* < 0.05) (Figure 3B). When it came to the material type, specific activity of PE showed significantly higher levels than PLA and glass, which confirmed that the material type could influence the bacterial specific activity on plastics. The BP of PE, PLA and glass in the average of the first two sampling dates (D3 and D10) were 1.9, 0.3, and 0.4 fgC cell^–1^ h^–1^, respectively, decreased to D30 (0.45, 0.15, and 0.20 fgC cell^–1^ h^–1^, respectively), and increased until D66 (2.5, 1.5, and 0.2 fgC cell^–1^ h^–1^, respectively). The material size and shape could also impact the specific activity, no difference was found between the size of 18 mm and irregular microplastic of 100 μm, while BP of the samples in 3 mm showed higher activity, and regular microbeads of 100 μm showing the lowest.

Accordingly, the bacterial growth rate on PE was higher than on PLA and glass. The bacterial growth rate of PE, PLA and glass had the average of the first two sampling dates (D3 and D10) with 3.8, 0.6, and 0.8 day^–1^, respectively, decreased to D30 (0.9, 0.3, and 0.4 day^–1^), and drastically increased until D66 (5.2, 3.0, and 0.5 day^–1^, respectively). The growth rate measured in seawater was 2.8, 6.4, 2.0, and 5.2 day^–1^ for D3, D10, D30, and D66, respectively.

### Diversity Indexes

Illumina MiSeq DNA sequencing generated 3 823 342 sequences tags, falling into 5293 ASVs after randomly resampling to 5177 sequences per sample to provide statistical robustness when comparing diversity measures among samples. DNA quantity were not sufficient to allow the sequencing of several samples at the early colonization stage (D3) that failed for glass samples including regular microbeads of 100 μm, 3 and 1.8 mm size, and also 3 mm for PE and PLA. A total of 123 samples were used for the following analyses, excluding also two samples (irregular PE microbeads and seawater) with low number of reads at D30. Rarefaction analysis suggested that all samples reached a stationary phase (data not shown).

**FIGURE 4:**
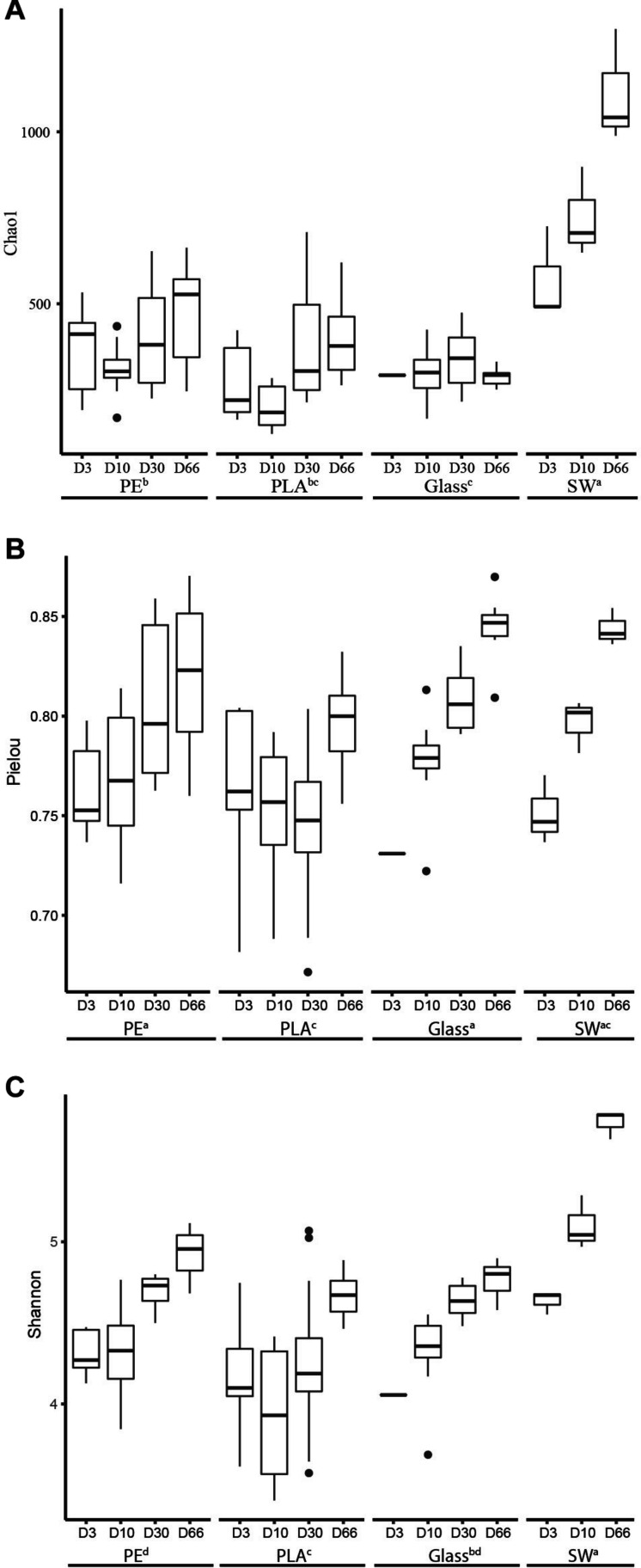
Alpha-diversity indices of **(A)** richness (Chao1), **(B)** evenness (Pielou), and **(C)** diversity (Shannon) of all the materials (PE, PLA, glass) and the surrounding seawater during the course of the experiment at days 3 (D3), 10 (D10), 30 (D30), and 66 (D66). The boxplots show the median between triplicate samples of different sizes for each material (heavy horizontal line inside the boxes), the box represents the first and third quartiles and unfilled circles indicate outliers. Lowercase letters (a, b, c) denote statistically different groups (*p* < 0.05).

Chao 1 estimator showed that the richness increased drastically in seawater during the course of the experiment, ranging on average from 570 to 1110 ASVs at D3 and D66, respectively. A slighter Chao 1 estimator increased was also found from D3 (average of 363, 265, and 267, respectively) to D66 (468, 395, and 288, respectively) for PE, PLA and glass, indicating that on the primo-colonization phase, a handful of bacterial species already colonizing the plastic surface (Figure 4A). Seawater sample showed significant higher Chao1 richness than PE, PLA, and glass. No difference was found on the Pielou index between seawater and PE, PLA or glass (Welch’s *p* > 0.05), where evenness increased for all materials and seawater samples at the end of the experiment (Figure 4B). Shannon diversity index of PE and glass was significantly higher than PLA (average of 4.6, 4.5, and 4.3, respectively) and no significant difference could be found in relation to the size of the different materials (Welch’s ANOVA test) (Figure 4C). High correlation of the temporal dynamic of Shannon index and Pielou evenness was found between 3 material types and seawater diversity (Pearson correlation, *p* = 0.018, *r* = 0.75; and *p* = 0.03, *r* = 0.71, respectively).

### Bacterial Community Structure

**FIGURE 5:**
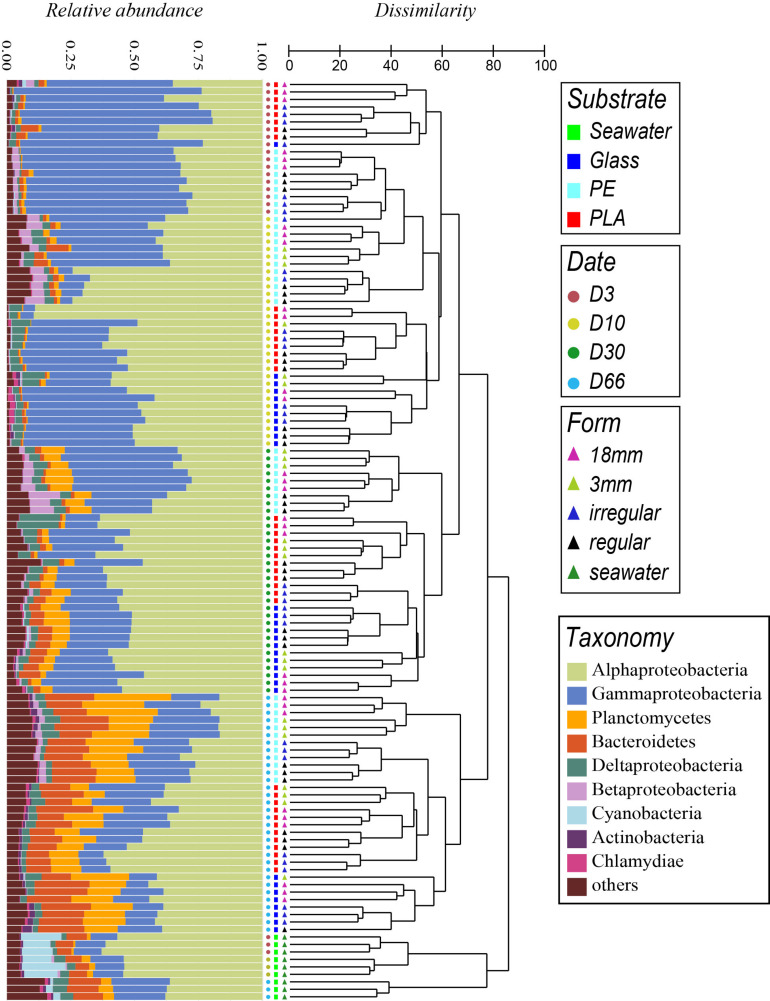
Comparison of temporal variation of taxonomic abundances and community structure of bacteria in seawater (SW) and biofilms of different materials (PE, PLA and glass) according to immersion time in days (D), by cumulative bar charts comparing relative abundances (left) and by UPGMA dendrogram based on Bray–Curtis dissimilarities between 16S rRNA-based sequencing profiles (right).

Unweighted-pair group method with arithmetic dendrogram based on Bray-Curtis dissimilarities showed that each of the triplicate samples in each sampling time and condition clustered together (except for one sample D10-PE-IR), confirming the homogeneity within the triplicates and also the proper sampling strategy, rigorous DNA extraction, sequencing and data processing (Figure 5).

Clear dissimilarities were found between bacterial communities in seawater and material types (PA, PLA, glass) (dissimilarity > 85%) and between samples corresponding to before (D3 to D30) and after the diatom bloom (D66) within each cluster (dissimilarity > 75%). Similarity profile testing (SIMPROF) showed these groups to be highly significant (*p* < 0.001).

Global PERMANOVA analyses with all samples confirmed the variance were highly explained by sampling date, to a less extent by chemical composition and material size (*R*^2^ = 0.39, 0.14, and 0.05 for PE, PLA, and glass, respectively, *p* < 0.01) (Supplementary Table 1). Pairwise PERMANOVA analyses confirmed that the factors of sampling date or material chemical composition can significantly explain the changes of bacterial community structure within each group, with higher values found between the primo-colonization or the growing phase compared to the mature biofilm influenced by the diatom bloom (*R*^2^ > 0.37, *p* < 0.01). Smaller but significant differences were found between substrate types (*R*^2^ = 0.07 between PLA and glass and *R*^2^ = 0.11 and 0.14 between PE and PLA or glass, respectively; *p* < 0.01), indicating that the bacterial community is more similar between PLA and glass than with PE. However, no significant difference between material size or shape could be found (*p* > 0.05), which was also supported by dispersion analyses (*p* > 0.05) (Supplementary Table 2).

Samples from the primo-colonization (D3) and the growing phases of the biofilms (D10) grouped in separated clusters for PLA and glass (dissimilarity 59%), and also for PE but with less dissimilarity among samples (48%). A clear shift in bacterial community was observed when the biofilms became mature (within D30 – dissimilarity 66%) followed by a drastic change after the diatom bloom (within D66 – dissimilarity 77%), whatever the material type (PE, PLA, glass) and size. All the above cited clusters were significantly different when using the SIMPROF tests (*p* < 0.05).

The time was the main factor driving the bacterial communities, the material type showed also different patterns. At each sampling date, PE formed always a specific cluster with all the size fractions. This was also the case for PLA and glass, except for the primo-colonization (D3) where they grouped in a same cluster.

Interestingly, the Mantel tests showed a significant relation between temporal changes in the seawater communities and in the biofilms growing on the different material types (Supplementary Table 3). Permutating correspondence between seawater and biofilms communities showed high correlation (Spearman rank = 0.84; *p* < 0.05) within dates (D3, D10, D66 seawater samples compared to biofilms at the same dates) that decreased drastically when permutating dates and for D66 in particular (Spearman rank < 0.5; *p* < 0.05). Shared ASVs between seawater and biofilms were <25% for PE, <17% for PLA and glass, but these ASVs were abundant in the samples since they represented >48% of the tags in all cases, and maximum reached at 62% for PE on D3 (Supplementary Figure 2). SIMPER analyses based on presence and absence data showed high dissimilarity among each substrate alongside the temporal evolution (61, 65, 63, and 60% for PE, PLA, glass, and seawater, respectively), while the shared ASVs and shared tags remained constant, suggesting that the bacterial community from the biofilm and seawater shift in the same direction.

### Taxonomic Composition

Taxonomic analyses confirmed the specificity of the community structures formed on the different materials compared to seawater, the latter being dominated by Alphaproteobacteria and composed mainly of Gammaproteobacteria, Cyanobacteria, Bacteroidetes, and Actinobacteria throughout the experimentation (Figure 5).

The distinct phases of biofilm formation were also clearly visible. The primo-colonization phase (D3) was dominated by Gammaproteobacteria (61 ± 0%, 61 ± 12%, and 71% for PE, PLA, and glass, respectively) and Alphaproteobacteria (31 ± 2%, 31 ± 9%, and 23% for PE, PLA and glass, respectively), dominant family were Alteromonadaceae from Gammaproteobacteria and Rhodobacteraceae from Alphaproteobacteria. The growing phase resulted in a decrease of Gammaproteobacteria (27 ± 18%, 29 ± 15%, and 40 ± 8% for PE, PLA, and glass, respectively) and a concomitant increase of Alphaproteobacteria (53 ± 17%, 64 ± 15%, and 51 ± 5% for PE, PLA, and glass, respectively). The main change for the maturation phase (D30) was the increase of Planctomycetes (8 ± 1%, 4 ± 2%, and 6 ± 2% for PE, PLA and glass, respectively) and of Bacteroidetes mainly for glass but not for PE and PLA (5 ± 2% for glass). At this stage, Gammaproteobacteria (39 ± 9%, 22 ± 5%, and 25 ± 5%) became as abundant or even less abundant as Alphaproteobacteria (34 ± 6%, 58 ± 5%, and 54 ± 4% for PE, PLA, and glass, respectively).

The mature biofilm changed drastically in the presence of diatoms (D66) with a continuous increase of Planctomycetes (20 ± 5%, 10 ± 5%, and 16 ± 3% for PE, PLA, and glass, respectively) and Bacteroidetes (16 ± 2%, 12 ± 3%, and 18 ± 3% for PE, PLA, and glass, respectively). The presence of diatoms at this stage was confirmed when looking at the eukaryote sequences that were initially removed for the bacterial diversity analysis. Note that the number of eukaryotic sequences increased at the end of the experiment (D66), especially for glass where eukaryotic sequences could reach until 19% of the total reads per sample, but also for PE and PLA where they can reach until 12 and 9%, respectively (data not shown). Interestingly, eukaryotic sequences were always <4% before the D66 on these materials and represented <0.7% all along the experiment in seawater. More than 50% and until 86% of the eukaryotic sequences belonged to the diatom *Pseudo-nitzschia* sp. on D66 for PE, PLA, and glass, whereas the sequences of *Pseudo-nitzschia* sp. in seawater remained relative very low (<10%).

**FIGURE 6:**
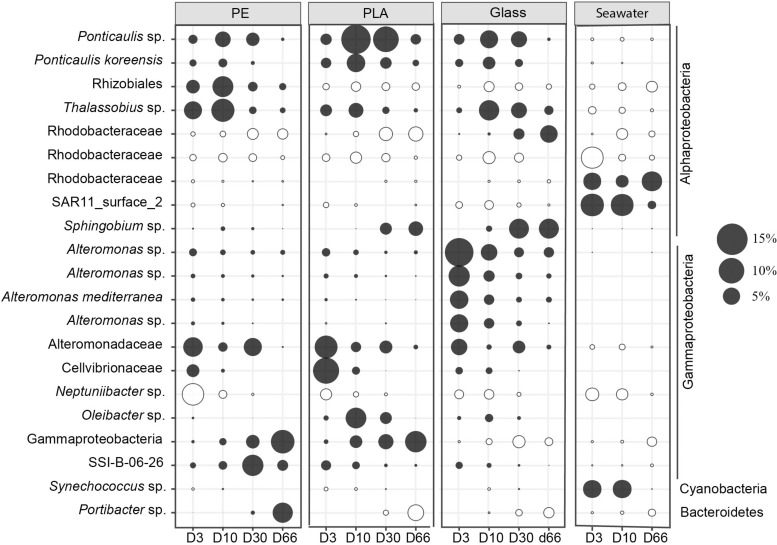
Bubble plot showing the relative abundance of the majority ASVs (>5%). Each dot corresponds to the average abundance among different size (18 mm, 3 mm, IR and RE). The closest taxonomic affiliation of each ASV was shown on the left of the panel. Black filled color indicates significant difference between seawater and different materials (PE, PLA, and glass) considering all sampling date (Welch’s ANOVA test).

We followed in particular 21 dominant bacterial ASVs that accounted for >5% of the sequences in each substrate for individual sampling date (Figure 6). Among those top 21 ASVs, 5 were unique for PE, PLA or glass that were not visible in seawater (Figure 6). We found the SAR11_surface_2 and ASV of Rhodobacteraceae more abundant in seawater compared to biofilms on the different material types.

During the primary colonization phase (D3), one ASV belonging to Alteromonadaceae was abundant in all material types, and distinction could be made between *Neptuniibacter* sp. and *Thalassobius* sp. that were more abundant on PE, whereas ASV of Cellvibrionaceae were more abundant in PLA, and *Alteromonas* sp. in glass samples. During the growing phase (D10), *Thalassobius* sp. were more represented on both PE and glass, while *Ponticaulis* sp. and *Oleibacter* sp. were more found for PLA. Changes between the maturation phase (D30) and the diatom bloom event (D66) varied according to the material types. The ASV of SSI-B-06-26 was more abundant on PE at D30 and ASV of Gammaproteobacteria and *Portibacter* sp. increased drastically at D66. *Ponticaulis* sp. dominated PLA at D30, while it switched to ASV of Gammaproteobacteria at D66. Less changes were found for glass where *Sphingobium* sp. remain abundant for glass both at D30 and D66.

## Discussion

Our results are providing the first complete description of bacterial abundance, diversity and activity during the three classical colonization phases (primo-colonization, growing and maturation phases) of biofilm formation on plastics. We also showed that the mature biofilm can be drastically modified by a phytoplankton bloom, thus indicating that environmental conditions may have a crucial role on shaping the plastisphere. Our experimental design enable us to evaluate the relative influence of plastic composition, size or shape as well as the importance of phytoplankton-bacteria interactions in shaping the plastisphere.

### Effect of Size and Shape of the Substrate and Its Chemical Composition on the Plastisphere

Raw measured values of abundance were found to differ between diverse size (meso-plastics of 18 mm diameter, LMP of 3 mm diameter and SMP of 100 μm diameter) and shapes (irregular IR and regular RE) for a given material. However, once the abundance was expressed per unit surface as in Figure 2, the differences disappeared between mesoplastics, LMP and SMP of RE or IR shapes. These results show, for the first time, that the apparent size effect on raw data is only due to the difference in specific surface (i.e., surface to volume ratio) for different particle shapes. For a same mass of material, an irregular surface has a larger available surface than a regular one, which therefore leads to a higher abundance hence a higher activity. In the present experiments, the excess surface is typically around 1.5 fold higher for IR shape compared to RE shape.

Besides, there was also no effect on the bacterial diversity from the different material size or shape. This result is in accordance with the only one study focusing on the effect of plastic size on bacterial diversity from coastal seawater (Brest, France), where no size effect was found between SMP (0.3 to 1 mm) and LMP (1 to 5 mm) (Frère et al., 2018). By contrast, our data showed dissimilarity in bacterial heterotrophic activities between different plastic size and shape. For instance, bacterial activity from PE in 3 mm (slight curly) was higher than other that of 18 mm, and bacterial activity from IR shape was higher than that of RE shape. This could be explained by very large roughness at the typical scale of a bacteria, one could expect in addition, a different packing of bacteria or a different biofilm structure – due for example to adhesion differences – which could induce differences in density of bacteria and/or in their heterotrophic production. In this study though, the roughness of all samples (including IR beads) was of the order of 100 nm over 100 × 100 squared microns areas, so that such effects -if any- would not be visible for the bacterial abundance or diversity. Other studies on roughness at a very small scale would need to be undertaken if one wants to conclude on this aspect. The present experiments also seem to show that in the present case, the temporal dynamic of biofilm formation together with the material type were always more important factors than the material size and shape for shaping the bacterial abundance, diversity and activity. However, it has been previously shown that the bacterial spatial position of natural plastisphere on particles is not totally even distributed and hence could depend on the size of the particles (Schlundt et al., 2019). Further studies with particle size less than the minimum size used in this study (100 μm) would probably be necessary to clarify this point.

During the three phases of its development, the biofilm evolved in a fairly different way on the two polymers studied (PE and PLA) and glass. PLA and glass were more similar in terms of bacterial activity and diversity compared to PE, while PE and PLA had similar and higher bacterial abundance compared to glass. In general, PE showed drastic differences compared to glass, while PLA showed intermediate values between them. The result is also pointing out that the bacterial activity could be not correlated to its abundance on plastisphere, which is similar to that found in seawater (Campbell et al., 2011). Our results are in accordance with another demonstration of significant influence of material type (PE, PLA, and Glass) on bacterial diversity (Kirstein et al., 2018). Our results tends to confirm the role of wetting properties: polymer surfaces being far more hydrophobic than glass. Attachment to surface are indeed supposed to be mediated via specific and non-specific interactions, both depending on surface hydrophobicity/hydrophilicity, roughness, charge and functional groups (Caruso, 2020). The role of hardness cannot, however, be excluded since it was recently shown to also be a key factor compared with other physicochemical properties (Cai et al., 2019).

Other factors may impact the plastisphere, such as the buoyancy: HDPE (0.95 g cm^–3^) was floating, whereas PLA (1.25 g cm^–3^) settled on tank bottom in our experimental conditions, which could lead to a different oxygenation of water or a different exposure to bacteria (composition, light intensity, contact frequency, exposition to air versus water). The buoyancy may explain the small but significantly higher average activity on PE compared to PLA, which differed also in term of community structure. Even though PLA is a compostable polymer and degradable in human body (Pillai and Sharma, 2010), it is known that PLA, like PE, is not biodegradable in marine environment (Karamanlioglu et al., 2017), and certainly not in the relatively short time frame of this experiment. Colonization, heterotrophic activity as well as the specificity of the species observed are therefore certainly not related to any biodegradation of the polymers. It seems more relevant to attribute the colonization, activity and specialization of bacteria to the formation of a conditioning film and subsequently the chemical composition of extracellular polymeric substance which could also be surface properties dependent.

### Presence of Three Following Phases of the Biofilm Development in All Substrates

Confocal microscopy was a powerful tool to follow the biofilm formation on the various samples tested in our study. For all studied material, the triplicate samples out of all the sampling dates highlighted three classical successive phases (primo-colonization, growth, and maturation) of biofilm formation. Such succession has already been observed on natural (rocks and algae) or artificial surfaces (glass, acryl, steel, and plastics) immerged in seawater (Caruso, 2020), such as PE sheets and dolly ropes during long-term exposure experiment in the North Sea (De Tender et al., 2017) or non-biodegradable and biodegradable plastics in the Mediterranean sea (Dussud et al., 2018a).

First, the primo-colonization designates the pioneer bacteria that shape the first layer of initial biofilm (Lorite et al., 2011). After 3 days of immersion in natural seawater, we observed that the primo-colonizers presented a higher abundance and heterotrophic activity (^3^H leucine incorporation) on PE compared to PLA and glass. MiSeq 16S rRNA sequencing revealed that bacterial richness was already high after 3 days with a minimum average Chao1 estimation of 265 ASVs whatever the material types. We observed distinct but closed bacterial communities in PE, PLA, and glass, dominated by Gammaproteobacteria (61, 61, and 71% for PE, PLA, and glass, respectively) and Alphaproteobacteria (31, 31, and 23% for PE, PLA, and glass, respectively). Previous studies showed that the *Roseobacter* clade and *Alteromonas* were the main bacterial primary colonizers (Dang and Lovell, 2000; Salta et al., 2013; Dang and Lovellc, 2016). From our results, we reported that *Thalassobius* sp. of the Roseobacter clade could be also the primary colonizer on plastisphere. *Thalassobius* sp. was also found on 3-day-old polymethyl methacrylate (PMMA) panel immersed the Arabian Gulf with relatively low abundance compared to our study (Abed et al., 2019) as well as on PE plastisphere from North Atlantic (Zettler et al., 2013). *Alteromonas* sp. was extremely abundant on glass samples compared to PE and PLA, where the three materials occupied different ASVs from Alteromonadaceae (Figure 5). *Neptuniibacter* sp. was also one of the main taxa revealed in our study, but also on 7-day-old colonized poly(3-hydroxybutyrate-co-3-hydroxyvalerate) (PHBV) from the Banyuls Bay, France (Dussud et al., 2018a). Even though it is considered that the *Neptuniibacter* sp. is an hydrocarbonoclastic bacteria (Dombrowski et al., 2016), we showed here that it might be just a primary colonizer, while not participating to the plastic degradation.

Second, the growing phase of the biofilm is described growth by secondary species, which induce modifications in the properties of the substratum (Lorite et al., 2011). After 10 days immersion in seawater, we have seen an increase in bacterial abundance and heterotrophic activity, together with significant changes in bacterial community structure for all material types. In particular, Alphaproteobacteria became more abundant (53, 64, and 51% for PE, PLA and glass, respectively) compared to Gammaproteobacteria (27, 29, and 40% for PE, PLA, and glass, respectively). *Thalassobius* sp. was still abundant in the growing phase after the primo-colonization phase. In addition, we firstly reported the *Ponticaulis* sp. as an important group of primary colonizers on plastisphere, strikingly on PLA. Besides, previous study also showed that *Ponticaulis* sp. was one of the main colonizers for the metallic alloys (Procópio, 2020). It suggested that the *Oleibacter* sp. could be one of the pioneer bacteria for plastic colonization within hours (Pollet et al., 2018), while we showed that it was more obvious on PLA in the growing phase after 10 days.

Third, the “maturation phase” occurs through diverse, competitive or synergistic interactions between cells, with either further recruitment or loss of species (Lorite et al., 2011). This has led to a stabilization of bacterial abundance and heterotrophic bacterial production, together with a drastic shift in bacterial community structure in all material types. PE still presented significantly higher abundance and activity compared to glass, whereas PLA showed similar bacterial abundance compared to PE and similar bacterial activity compared to glass. At this stage, Gammaproteobacteria (39, 22, and 25%) were as abundant or even less abundant as Alphaproteobacteria (34, 58, and 54% for PE, PLA, and glass, respectively). Bacteroidetes and Planctomycetes were also found as secondary colonizers in other studies (Dang and Lovellc, 2016; Pinto et al., 2019). We observed noticeable increases of Planctomycetes (8, 4, and 6% for PE, PLA, and glass, respectively) and also Bacteroidetes (mainly for glass (5%) but not for PE and PLA). Interestingly, we observed a similar evenness associated to an increase of richness during the growing and maturation phases, which is characteristic abundant and heterogeneous resources and nutrients offered by plastic particles compared to the nutrient-depleted oceanic deserts (Zhou et al., 2002).

During this study, we found that the Roseobacter and Alteromonas were important clades for whatever the three-colonization phases, while the two clades were also found as bacterial “phycosphere” (bacterial taxa colonizing on phytoplankton) (Seymour et al., 2017). Thus, we suspect that the plastic surface and phytoplankton surface could have some similar trait to be the environmental cue for these two clades. Another long term study identified the consistent presence of *Polaribacter sp.*, *Kangiella sp.*, *Lacinutrix sp*. during a 44 weeks experiment using plastic sheet and dolly rope immerged at an offshore station in the North Sea (De Tender et al., 2017). These taxonomic groups were dissimilar from the persistent taxa observed in our study, probably because of difference between the plastic substrates and the environmental conditions.

### Influence of Phytoplankton Bloom on the Mature Biofilm

In our study, the diatoms were presented in seawater during the entire course of the experiment course, while a diatom bloom was observed at D66 on plastisphere. It suggested that a bacterial biofilm would be a prerequisite for the diatom bloom on plastisphere. Most of the microalgae sequences on plastisphere belonged to *Pseudo-Nitzschia* sp., which was in accordance to the observation of their typical morphotype on D66 with confocal microscopy technique on all material surfaces. The diatoms were more observed on the film other than small microplastics, suggesting that the rigidity morphology of diatoms require more flatter surface area for the colonization comparing to bacteria. *Pseudo-nitzschia* is a global distributed diatom genus in the marine environment (Lelong et al., 2012). It has not only been reported in the Mediterranean Sea of marine observatory stations in the Banyuls Bay (France), but also in the 150 km-south Blanes Bay (Spain) where the phytoplanktonic bloom in seawater were consistently attributable to chromophytes, the most abundant taxa being *Pseudo-nitzschia calliantha* (Mura and Agustí, 1996; Charles et al., 2005).

Diatoms have been found as omnipresent and sometimes dominant colonizers on plastic debris (Oberbeckmann et al., 2014; Maso et al., 2016; Michels et al., 2018; Kettner et al., 2019). Morphological identification by microscopy together with new chloroplast databases from bacterial amplicon surveys (Decelle et al., 2015) included *Mastogloia*, *Cyclotella*, *Pleurosigma, Amphora*, and *Pseudo-Nitzschia* genera in the Arabian Gulf, Grenada Island, Atlantic, and Pacific gyres (Amaral-Zettler et al., 2020). *Pseudo-nitzschia* spp. has been identified as the dominant diatoms on 10-day-old biofilm developed on polystyrene Petri dishes immersed at the low intertidal zone, Hong Kong (China) (Chiu et al., 2008). To our knowledge, it is the first time that *Pseudo-nitzschia* spp. were identified as dominant phototrophs on plastic debris on Mediterranean plastisphere. The diatoms event happened on plastisphere could be also related to the diatom bloom events happened on Banyuls Bay (Supplementary Figure 1C).

Interaction between phytoplankton and bacteria are known to play key roles in mediating biogeochemical cycling and the food web structure in the ocean, including the microbial loop (Mayali, 2018). Diatom blooms are also known to be one of the main drivers of the temporal dynamics of bacterial abundance, diversity and activity in the Mediterranean Sea and elsewhere (Ghiglione et al., 2005; Lambert et al., 2019), with consistent taxonomic association between specific bacteria and diatom taxa (Behringer et al., 2018). Our results confirm that such association exist also within the biofilms associated with plastic, as it has been observed in other studies (Amaral-Zettler et al., 2020). We found co-associated bacterial epibionts on the mature biofilms at D66 that were related to the specific biofilm of each polymer type. For example, we found common colonizers of diatom detritus, such as *Portibacter* sp. (Crenn et al., 2018), and *Sphingobium* sp. (Ramanan et al., 2015) and Rhodobacteraceae (previously mostly assigned as Roseobacter clade) (Simon et al., 2017). The interaction between diatoms and bacteria within the mature biofilms was accompanied with a drastic increase of bacterial heterotrophic activities in PE and PLA. This is a typical response of nutrient recycling heterotrophs to primary producing photoautotrophs, where bacterial activity per cell increases drastically together with changes in community structure (Mayali, 2018). Our results showed that the diversity and activity of the mature biofilms on plastic can be rapidly and drastically changed due to phytoplanktonic growth on plastics, whatever the polymer type, size or shape. To our knowledge, only one study so far measuring chlorophyll *a* and net primary production in the North Pacific gyre showed that microplastic particles were creating net autotrophic hot-spots in the oligotrophic ocean (Bryant et al., 2016). In parallel, another unique study in the Mediterranean Sea revealed higher bacterial heterotrophic activity on plastic compared to the surrounding seawater (Dussud et al., 2018a).

During this study, we cannot really compare the bacterial activity or growth rate between plastisphere vs. our seawater samples, because of the potentially lower bacterial abundance numeration on seawater samples. Instead, we propose the following scenario: the bacterial growth rate on the primo-colonization and growing phase should be higher than that of seawater, at least on PE samples, as previous study showed that the Roseobacter and Alteromonadaceae have relative high growth rate compared to the bulk bacterial community (Ferrera et al., 2011). The bacterial activity or growth rate on the maturation phase in the marine environment could be higher than seawater considering that autotroph microbes such as diatoms were omnipresent on plastics. Further works coupling both primary and heterotrophic production measurements are needed to determine the bacterial activity difference between plastisphere and seawater, but also test if the microscale algal-bacterial interactions on the large amount of plastic floating in sea surface have consequences on ecosystem functioning and/or biogeochemical cycling.

### Concluding Remarks

Here we prove that phytoplankton-bacteria interactions may greatly modify the plastisphere, which can no longer be considered as a vector of a durable and stable community, but rather a vector of communities interacting with their environment and subjected to changes. We showed that phytoplankton-bacteria interactions do not influence the abundance of the mature biofilm formed on plastics, but may drastically impact the diversity and the heterotrophic activities of the plastisphere. These results may have consequences in the further evaluation of the functional role of the plastisphere, such as its contribution to the biogeochemical cycles of elements. Another major finding of our study was that the size and the shape of the plastics showed little influence on the plastisphere abundance, diversity and activity, which is in accordance with the few studies dealing with this aspect but focusing on the diversity only. Hydrophobicity, topography, roughness, crystallinity, and surface charge have been previously found to influence the early stage of plastic colonization, whereas the plastisphere directly sampled at sea was found to be rather driven by geographical location or seasons, which are typically related to environmental conditions. Our results, together with the growing literature in this field, are opening the description of the complex and fascinating interactions between the plastisphere and its plastic support, but also with the surrounding environment that may greatly influence its spatial and temporal dynamics.

## Data Availability Statement

The datasets presented in this study can be found in online repositories. The names of the repository/repositories and accession number(s) can be found below: https://www.ncbi.nlm.nih.gov/genbank/, PRJNA663787.

## Author Contributions

JC, A-LM, and J-FG: conception and design of study. JC, JJ, and VB: acquisition of data. JC, MG, PF, A-LM, and J-FG: analysis and interpretation of data. SB, AT, A-LM, and J-FG: provision of equipment. JC and J-FG: drafting the manuscript. PF, MG, SB, AT, and A-LM: revising the manuscript critically for important intellectual content. JC, JJ, PC, MP-P, VB, MG, PF, SB, AT, A-LM, and J-FG: approval version of the manuscript to be published. All authors contributed to the article and approved the submitted version.

## Conflict of Interest

The authors declare that the research was conducted in the absence of any commercial or financial relationships that could be construed as a potential conflict of interest.

## Footnotes

1 http://somlit.epoc.u-bordeaux1.fr/
