# Supplementary material for: Relative Influence of Plastic Debris Size and Shape, Chemical Composition and Phytoplankton-Bacteria Interactions in Driving Seawater Plastisphere Abundance, Diversity and Activity
Source: Front Microbiol. 2021 Jan 13;11:610231. doi: 10.3389/fmicb.2020.610231 (PMC7838358; doi:10.3389/fmicb.2020.610231)
Supplement: Supplementary file 1 [file Data_Sheet_1.PDF]

Table S 1: Global PERMANOVA on the factor of sampling date, chemical composition and material size

	Df	Sum of square	Mean of square	F value	R^2	Pr(>F)	p.betadisper
Size	3	1.2434	0.41447	1.842	0.04783	**0.011**	0.05165
chemical composition	2	3.5863	1.79314	8.8822	0.13796	**0.001**	0.4128
sampling date	3	10.149	3.3828	23.483	0.3904	**0.001**	**0.0001397**
Total	113	25.995			1.0000		

Df: degree of freedom. Note that: except for seawater, all the rest of samples were used for the analyses of the global PERMANOVA. The size fraction include the 100 µm, 3mm and 18 mm, where the size fraction of the 100 µm had two material forms of the irregular and regular shapes. Chemical composition included the PE, PLA and glass. Sampling date included the Day 3 (D3), D10, D30 and D66.
