# Supplementary material for: Relative Influence of Plastic Debris Size and Shape, Chemical Composition and Phytoplankton-Bacteria Interactions in Driving Seawater Plastisphere Abundance, Diversity and Activity
Source: Front Microbiol. 2021 Jan 13;11:610231. doi: 10.3389/fmicb.2020.610231 (PMC7838358; doi:10.3389/fmicb.2020.610231)
Supplement: Supplementary file 2 [file Data_Sheet_2.PDF]

Table S 2: Pairwise PERMANOVA on the factor of sampling date, chemical composition and material size

	Pairs	R^2	*p*.value	*p*.adjusted	*p*.betadisper
Sampling date	D10 vs D3	0.155	**0.001**	**0.001**	**0.027**
	D10 vs D30	0.207	**0.001**	**0.001**	0.961
	D10 vs D66	0.375	**0.001**	**0.001**	**0.020**
	D3 vs D30	0.308	**0.001**	**0.001**	**0.027**
	D3 vs D66	0.416	**0.001**	**0.001**	**0.001**
	D30 vs D66	0.236	**0.001**	**0.001**	**0.020**
Chemical composition	Glass vs PE	0.138	**0.001**	**0.001**	0.968
	Glass vs PLA	0.068	**0.001**	**0.001**	0.432
	PE vs PLA	0.109	**0.001**	**0.001**	0.432
Material size	18mm vs 3mm	0.029	0.128	0.140	0.184
	18mm vs IR	0.031	**0.049**	0.122	0.081
	18mm vs RE	0.023	0.140	0.140	0.081
	3mm vs IR	0.054	**0.009**	0.054	0.774
	3mm vs RE	0.035	0.061	0.122	0.774
	IR vs RE	0.025	0.140	0.140	0.901

IR: irregular shape of the 100 µm; RE: regular shape of the 100 µm. Note that the seawater samples were excluded for the analyses.
