# Supplementary material for: Relative Influence of Plastic Debris Size and Shape, Chemical Composition and Phytoplankton-Bacteria Interactions in Driving Seawater Plastisphere Abundance, Diversity and Activity
Source: Front Microbiol. 2021 Jan 13;11:610231. doi: 10.3389/fmicb.2020.610231 (PMC7838358; doi:10.3389/fmicb.2020.610231)
Supplement: Supplementary file 3 [file Data_Sheet_3.PDF]

Table S 3: Mantel test between bacterial community from biofilm and seawater community.

	Correspondence	Spearman correlation (Rho)	Note
Biofilm community	D3, D10, D66	0.845*	No exchange[#]
Seawater community	D3, D10, D66		
Biofilm community	D3, D10, D66	0.2727*	exchange of D10 and D66[†]
Seawater community	D3, D66, D10		
Biofilm community	D3, D10, D66	0.7227*	exchange of D3 and D10
Seawater community	D10, D3, D66		
Biofilm community	D3, D10, D66	0.4511*	exchange of D3, D10 and D66
Seawater community	D10, D66, D3		
Biofilm community	D3, D10, D66	0.4583*	exchange of D3, D10 and D66
Seawater community	D66, D3, D10		
Biofilm community	D3, D10, D66	0.4975*	exchange of D3 and D66
Seawater community	D66, D10, D3		

Biofilm community contained all the samples of PE, PLA and glass except for the D30. Notes that seawater community permutation were taken before Mantel test for some groups. for instance, the symbol of # indicates no permutation. † indicates that one matrix of seawater community was permutated before the Mantel test for D10 and D66. * indicating p value less than 0.05.

From the results one can note that the correlation is the highest for the group without permutation, that means that the bacterial community from plastisphere and seawater were highly linked to each other.
