# Supplementary figures and images for: Relative Influence of Plastic Debris Size and Shape, Chemical Composition and Phytoplankton-Bacteria Interactions in Driving Seawater Plastisphere Abundance, Diversity and Activity
Source: Front Microbiol. 2021 Jan 13;11:610231. doi: 10.3389/fmicb.2020.610231 (PMC7838358; doi:10.3389/fmicb.2020.610231)

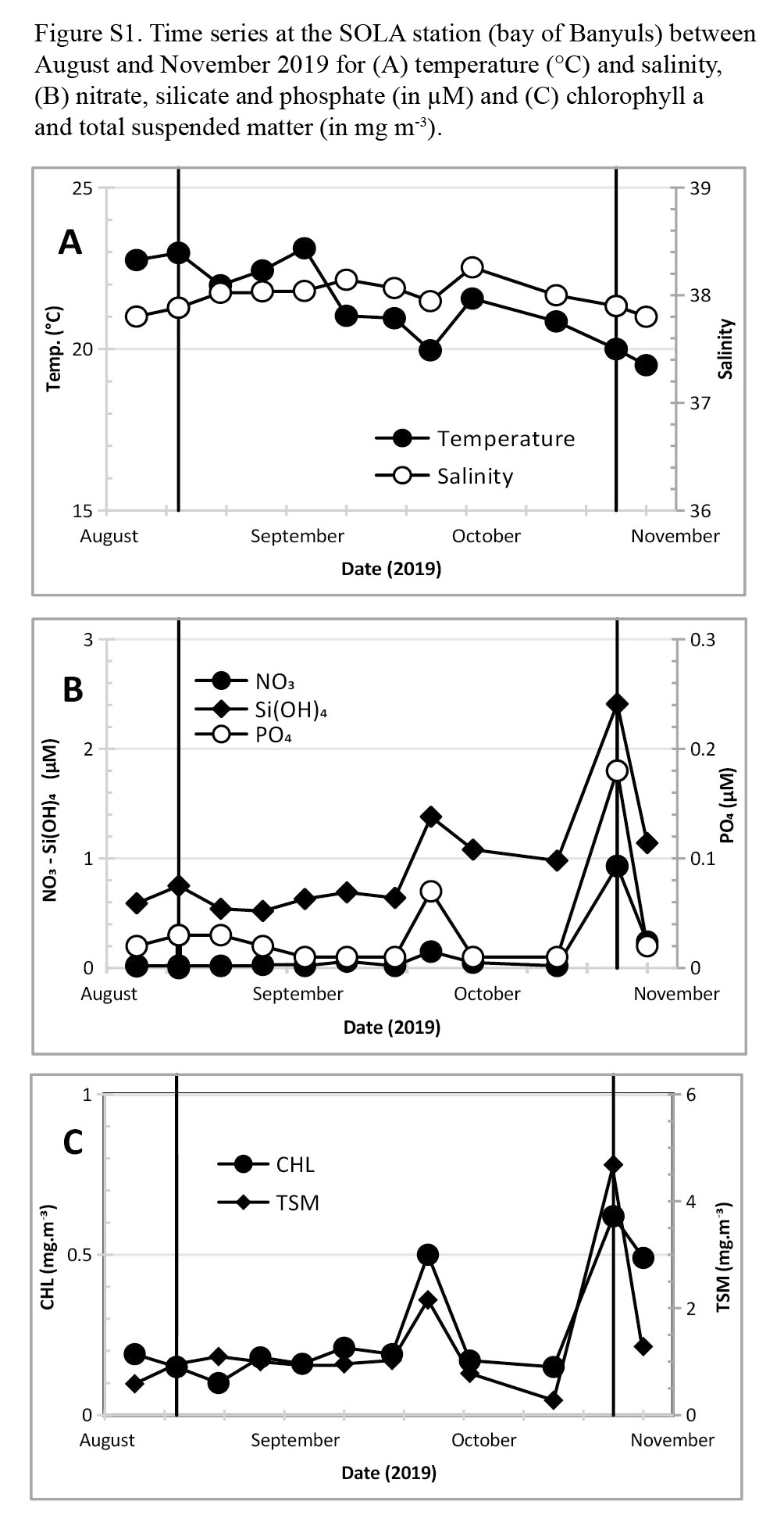

Supplement: Supplementary file 4 [file Image_1.JPEG]

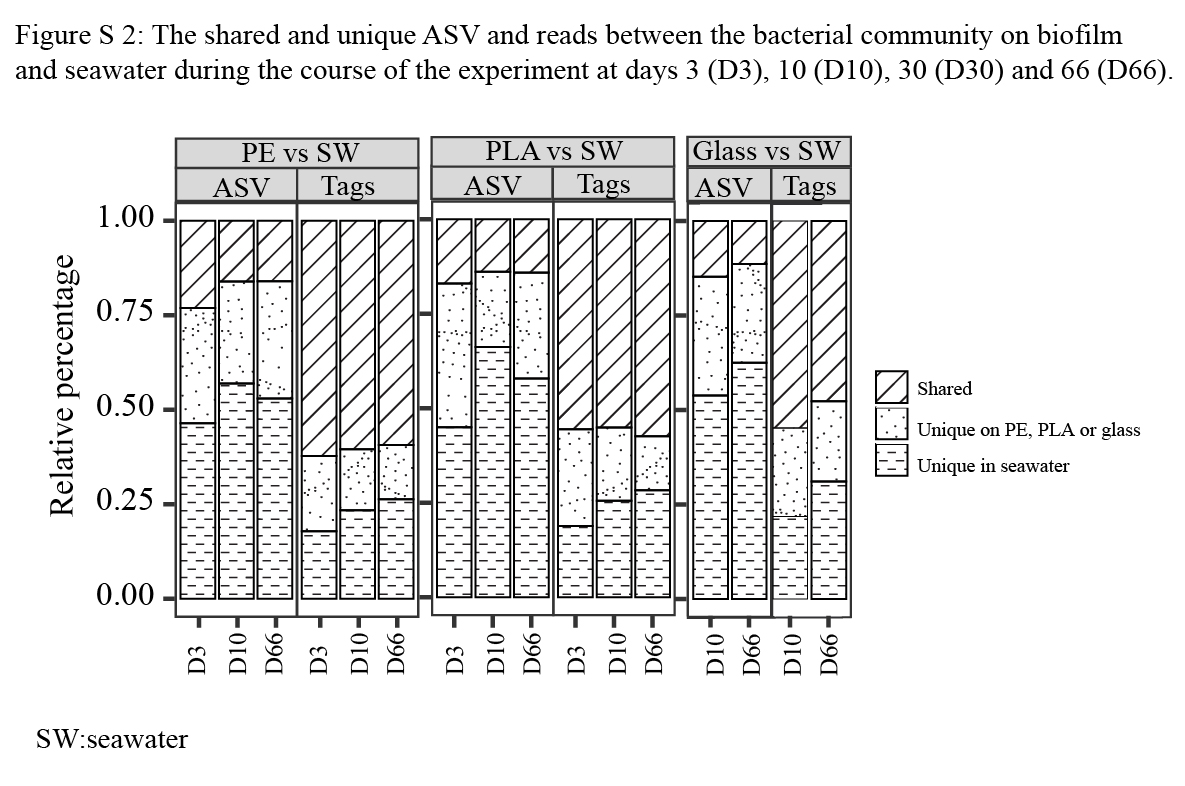

Supplement: Supplementary file 5 [file Image_2.JPEG]
